# Human papillomavirus and cervical cancer in the microbial world: exploring the vaginal microecology

**DOI:** 10.3389/fcimb.2024.1325500

**Published:** 2024-01-25

**Authors:** Zhemei Zhang, Qingmei Ma, Lei Zhang, Li Ma, Danni Wang, Yongqing Yang, Pengxia Jia, Yang Wu, Fang Wang

**Affiliations:** ^1^ Department of Clinical Laboratory, Gansu Provincial Hospital, Lanzhou, Gansu, China; ^2^ Gansu Provincial Clinical Research Center for Laboratory Medicine, Lanzhou, Gansu, China

**Keywords:** dysbiosis, probiotics, microbiota transplantation, biomarkers, personalized testing

## Abstract

The vaginal microbiota plays a crucial role in female reproductive health and is considered a biomarker for predicting disease outcomes and personalized testing. However, its relationship with human papillomavirus (HPV) infection and cervical cancer is not yet clear. Therefore, this article provides a review of the association between the vaginal microbiota, HPV infection, and cervical cancer. We discuss the composition of the vaginal microbiota, its dysbiosis, and its relationship with HPV infection, as well as potential mechanisms in the development of cervical cancer. In addition, we assess the feasibility of treatment strategies such as probiotics and vaginal microbiota transplantation to modulate the vaginal microbiota for the prevention and treatment of diseases related to HPV infection and cervical cancer. In the future, extensive replication studies are still needed to gain a deeper understanding of the complex relationship between the vaginal microbiota, HPV infection, and cervical cancer, and to clarify the role of the vaginal microbiota as a potential biomarker for predicting disease outcomes, thus providing a theoretical basis for personalized testing.

## 1 Introduction

Cervical cancer (CC) is the most common malignant tumor in the female reproductive system and a significant global public health concern that seriously threatens women’s health. Despite the implementation of human papillomavirus (HPV) vaccination and early screening for CC in reproductive-age women in most countries to prevent its occurrence, CC remains the fourth leading cause of cancer-related deaths in women. The GLOBOCAN 2020 database reported 604,000 new cases and 342,000 deaths worldwide (Sung et al., 2021), showing an increasing trend in both incidence and mortality compared to the 2018 global CC statistics (Bray et al., 2018).

It is well known that the development of CC is closely associated with HPV infection. HPV is primarily transmitted through direct contact with lesions of infected individuals or indirectly through contact with virus-contaminated objects. Newborns can also acquire HPV through birth canal infections. HPV mainly affects the skin and mucous membranes, causing varying degrees of proliferative lesions, and plays a significant role in the development of genital warts, cervical precancerous lesions, and CC (Burchell et al., 2006; Koshiol et al., 2008). Most women are infected with one or more types of HPV at least once in their lifetime, but the majority of these infections are transient and can self-clear within 1-2 years. Only a small fraction of women may sustain infections and have an increased risk of developing cervical precancerous lesions or invasive CC under certain influencing factors (Sudenga and Shrestha, 2013; Brusselaers et al., 2019). However, the persistence and clearance of HPV may be related to the vaginal microbiota (Zeng et al., 2022).

The female reproductive system has its specific microbial communities that play essential roles in women’s life processes and menstrual cycles (Chen et al., 2017), primarily maintaining vaginal health and protecting the vaginal environment from various genitourinary infections. The vaginal microbiota (VMB) interacts dynamically with the host and the environment, forming a dynamic ecosystem known as the vaginal microbiome, mainly maintained through interactions with the local microenvironment (Sharifian et al., 2023).

Research indicates that HPV is a necessary factor for CC and its precancerous lesions but not the sole determinant of CC development (Walboomers et al., 1999), suggesting that HPV alone is insufficient to induce cervical malignancy. Furthermore, the driving factors behind the transition states between HPV acquisition, clearance, persistence, and progression to cervical precancerous lesions remain unclear. However, some scholars have proposed that the VMB plays a significant role in the progression of cervical lesions induced by HPV infection (Gillet et al., 2011; Gillet et al., 2012; Audirac-Chalifour et al., 2016; Mitra et al., 2016; Brusselaers et al., 2019; Santella et al., 2022). Therefore, this paper reviews the impact of VMB on CC resulting from HPV infection, elucidating the role of VMB as a potential biomarker for predicting disease outcomes and providing a basis for personalized testing.

## 2 Healthy vaginal microbiome

The VMB plays a crucial role in protecting the host from infectious diseases and is considered a useful biomarker for predicting disease outcomes and personalized testing (Kyrgiou and Moscicki, 2022). A significant characteristic of a healthy vaginal microenvironment is the abundant colonization and dominance of *Lactobacillu*, primarily consisting of *Lactobacillus gasseri* (*L. gasseri*), *Lactobacillus crispatus* (*L. crispatus*), *Lactobacillus iners* (*L. iners*), *Lactobacillus jensenii* (*L. jensenii*), or *Lactobacillus vaginalis* (Younes et al., 2018; Chee et al., 2020; Fan et al., 2021). The composition of VMB varies during different life stages, including infancy, puberty, pregnancy, and menopause, due to hormonal changes, metabolic deposits, and antibiotic usage, with host estrogen levels having a significant impact on the vaginal environment (Das et al., 2023). Additionally, *Lactobacillus* spp. has a relatively low correlation with high risk-HPV (HR-HPV), cervical intraepithelial neoplasia (CIN), and CC (Wang et al., 2019).

### 2.1 Community state types typing

To describe the presence of different VMB profiles that may affect the development of cervical diseases, researchers have categorized them into Community State Types (CSTs). Using 16S rRNA gene amplicon sequencing, the VMB has been divided into five different CSTs: *L. crispatus* (CST I), *L. gasseri* (CST II), *L. iners* (CST III), low-*Lactobacillus* and bacterial vaginosis-associated bacteria (CST IV), and *L. jensenii* (CST V) (Ravel et al., 2011; Gajer et al., 2012; Mancabelli et al., 2021). CST IV is characterized by the augment in VMB diversity, mainly marked by a decrease in *Lactobacillus* abundance and an increase in anaerobic bacterial species. The most common bacteria in CST IV include *Gardnerella vaginalis*, *Megasphaera*, *Sneathia*, and *Prevotella* species (Castanheira et al., 2021).

The composition of VMB is dynamic and continuously changes in reproductive-age women. In healthy women, CST I and CST V are predominant. However, when infected with HPV, CST II significantly increases and promotes HPV clearance (Brotman et al., 2014b; Xu et al., 2020). A meta-analysis that examined the relationship between different CSTs and HPV infection showed that compared to *L. crispatus*, CSTs predominantly composed of ‘low *Lactobacilli*’ or *L. iners* had a 3-5 times higher risk of HPV infection and a 2-3 times higher risk of HR-HPV, cervical dysplasia, and CC occurrence (Norenhag et al., 2020). This suggests that VMB composition may potentially serve as a biomarker for HPV-related diseases, guiding clinical treatment.

### 2.2 Lactobacillus

*Lactobacillus* play a dominant role in the vaginal microbiota and contribute to maintaining vaginal microbiota balance, inhibiting the growth of pathogens, enhancing local vaginal immunity, and resisting tumors (Cheng et al., 2020).

**Figure 1:**
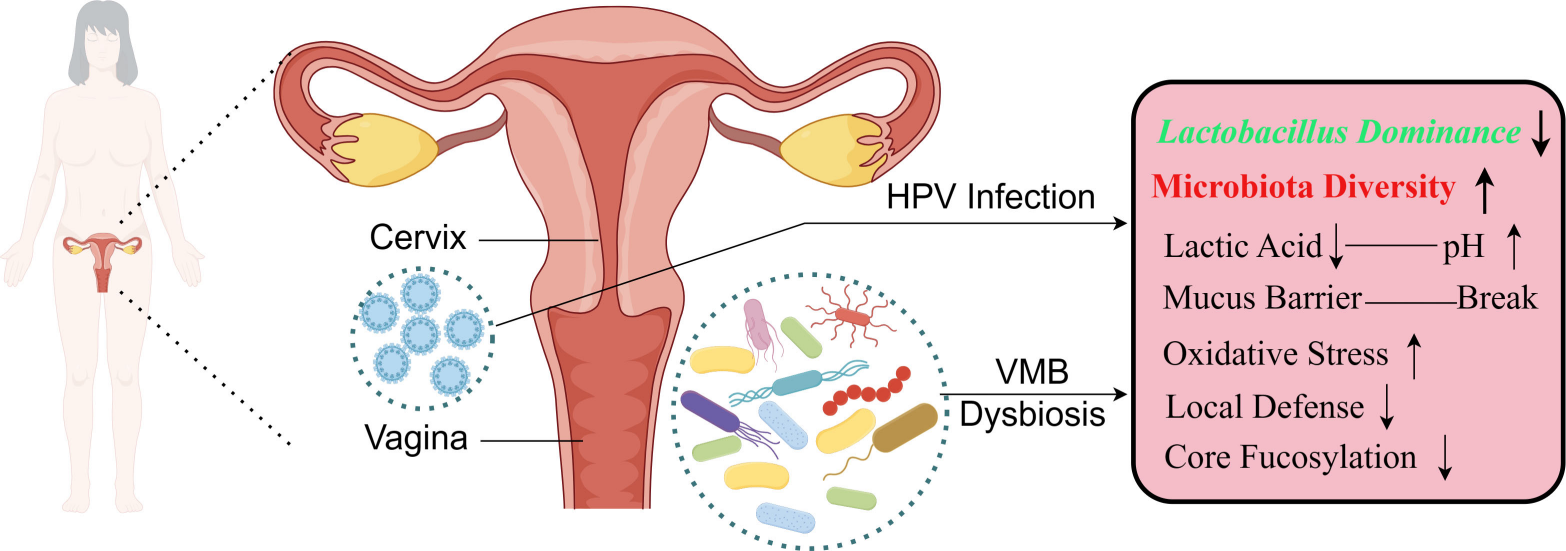
The alterations of the vaginal microenvironment when the dysbiosis of the VMB and HPV infection occur with cervical lesions.

The mechanisms through which *Lactobacillus* maintains the health of the female reproductive system include: (1) Lactic acid production and maintenance of vaginal acidity. *Lactobacillus:* primarily utilize carbohydrates on the vaginal mucosal epithelial cells as an energy source to produce lactic acid, maintaining the vaginal environment in a relatively acidic state. This inhibits the adhesion, colonization, and growth of pathogenic bacteria (Anahtar et al., 2018; Kroon et al., 2018). Additionally, L-lactic acid under acidic conditions can induce anti-inflammatory responses in cervical and vaginal epithelial cells while inhibiting the production of pro-inflammatory cytokines and chemokines induced by toll-like receptors (TLRs) (Delgado-Diaz et al., 2019). (2) Destruction of essential epithelial proteins for vaginal barrier integrity by bacteriocins and H_2_O_2_. Myeloperoxidase (MPO), an enzyme expressed in neutrophils, catalyzes the production of hypochlorous acid in the presence of H_2_O_2_ produced by *Lactobacillus*, preventing the invasion of pathogens and HPV into cervical epithelial cells (Castelão et al., 2015). Furthermore, *Lactobacillus* produce protective proteins such as biosurfactants and bacteriocins, disrupting epithelial cells and forming the first line of defense against pathogen adherence in the vaginal environment (Zevin et al., 2016; Borgogna et al., 2020; Nieves-Ramírez et al., 2021). (3) Competition with pathogens for vaginal epithelial adhesion due to steric hindrance or specific obstruction at the receptor site. *Lactobacillus* compete for space and resources with other bacteria, either promoting or preventing the colonization of other bacteria. Vaginal mucus and epithelial cell receptors may play important roles in the colonization of certain bacteria (Hickey et al., 2012). (4) Regulation of local defense. Regulating VMB balance and enhancing local cervical immune function may reduce the occurrence of cervical lesions (Zheng et al., 2020). (5) Regulation of core fucosylation of vaginal mucosal epithelial cells. Fucosylation of mucosal epithelial cells is closely related to microbial colonization (Goto et al., 2016). Knocking out the core fucosyltransferase gene (Fut8) can promote CC proliferation and migration, while the lactic acid produced by *L. iners* activates the Wnt pathway through the lactic acid-gp81 complex, increasing epithelial cell fucosylation levels, and inhibiting CC proliferation and migration (Fan et al., 2021). (6) Autophagy in infected cells and promotion of clearance (**Figure 1**).

### 2.3 Factors influencing vaginal microbiota

The normal function of VMB is influenced by various factors, including ethnicity, genetic background, epigenetic changes, multiple pregnancies, lifestyle, hygiene habits, infections, antibiotic use, age at first sexual intercourse, number of sexual partners, smoking, and long-term use of contraceptives and hormonal medications (Busnelli et al., 2019; Kaur et al., 2020). Ravel et al. found that in Asian and white women, *Lactobacillus*-dominated VMB occurred in 80.2% and 89.7% of cases, respectively, while in black and Hispanic women, it occurred in 59.6% and 61.9% of cases, respectively (Ravel et al., 2011). This is consistent with the results of Xia Zhou et al., indicating potential differences in the quantity and types of VMB among women of different ethnicities (Zhou et al., 2007). Furthermore, a meta-analysis suggests that vaginal douching may increase vaginal bacterial diversity, potentially increasing the risk of CIN and CC (Zhang et al., 1997). Additionally, research has found that smoking (Brotman et al., 2014a) and sexual intercourse (Mändar et al., 2015) can decrease the abundance of *L. crispatus* and increase microbiota diversity. However, oral hormonal contraceptives may disrupt host hormone levels, leading to changes in the vaginal environment (Das et al., 2023). Dysbiosis in the VMB can lead to the overgrowth of opportunistic pathogens, ultimately leading to disease development.

## 3 HPV and cervical cancer

HPV is a class of small, non-enveloped, double-stranded circular DNA viruses. The viral particles have an icosahedral shape with a diameter of 50-55 nm and a molecular weight of 5 × 10 ^6^ Da (Araldi et al., 2018). The HPV genome comprises 8 protein-encoding genes organized into three regions: (1) Non-coding region (NCR), containing the long control region (LCR) with promoters, enhancers, and silencers. This region is the least conserved segment of the HPV genome and is often used to describe relationships between variants. (2) Early region, including genes E1, E2, E4, E5, E6, and E7, which are involved in virus replication and transformation. (3) Late region, encoding the major (L1) and minor (L2) capsid proteins, participating in virus particle assembly (Tommasino, 2014; Sharifian et al., 2023). E1 is the initiation recognition protein for viral DNA replication. E2 is involved in viral DNA replication and transcription and can suppress the expression of E6 and E7. E4 interacts with the cellular cytoskeleton in the later stages of the virus lifecycle, and E5 may have a role in genome amplification. E6 and E7 mainly interfere with critical cell cycle checkpoints, such as inhibiting tumor protein p53 and retinoblastoma protein pRb (Haręża et al., 2022; Sharifian et al., 2023), forcing different epithelial cells to re-enter the cell cycle to increase virus production, thereby mediating HPV carcinogenesis (Pyeon et al., 2009; Lin et al., 2020). Recent literature reports that HPV E7 significantly inhibits the expression of host defense peptides, ultimately leading to VMB dysbiosis (Lebeau et al., 2022). Therefore, the expression of HPV E6 or E7 may be regulated by certain pathogens at different stages of cervical lesions (Liu et al., 2022).

Currently, more than 200 different HPV subtypes have been identified based on at least 10% sequence differences in the L1 gene sequence. Additionally, HPV is classified into low-risk types (LR-HPV) and HR-HPV based on the host diseases or cancers they cause. LR-HPV is responsible for mild benign proliferative lesions, while HR-HPV is more likely to lead to malignant changes. Among HR-HPV genotypes, HPV 16 and 18 are the most common and closely associated with the development of CC (Muñoz et al., 2006). S. Andersson et al. conducted a genetic typing of 131 cervical adenocarcinoma patients, and the results showed that HPV 18 and 16 had the highest infection rates, at 52% and 33%, respectively (Andersson et al., 2001). Furthermore, data indicates that out of 690,000 cancer cases caused by HPV infection, 570,000 were CC, with HPV 16 and 18 accounting for 72%, and HPV 31, 33, 45, 52, and 58 accounting for 17% (De Martel et al., 2020). This suggests that HPV 18 and 16 infections are major risk factors for cervical lesions.

Cervical lesions can be categorized into CIN I, II, and III based on the severity of cervical dysplastic cells (Brianti et al., 2017). Additionally, according to the Bethesda system, cervical precancerous lesions can be classified as low-grade squamous intraepithelial lesion (LSIL) and high-grade squamous intraepithelial lesion (HSIL) (Ostör, 1993). While HPV infection is a necessary condition for CC, it is not the sole factor. Other contributing factors may include the number of sexual partners, pregnancy history, genetic background, epigenetic changes, immune system status, ethnicity, and VMB imbalance (Lin et al., 2015; Boda et al., 2018).

Recently, a cross-sectional study found differences in vaginal metabolites such as biogenic amines, glutathione, and lipids between women with cervical HPV positivity and those with HPV negativity. If a correlation between the incidence/persistence of HPV and reduced glutathione or oxidative glutathione can be demonstrated, antioxidant therapy may become a non-surgical intervention for HPV (Borgogna et al., 2020).

## 4 Vaginal microbiota, HPV, and cervical cancer

In recent years, research has shown that VMB can modulate HPV infection and play a role in the development of cervical lesions (**Figure 1**). The composition of VMB may act as a regulator of HR-HPV (Chao et al., 2019). Increased diversity in VMB and reduced abundance of *Lactobacillus*, leading to vaginal dysbiosis, may be associated with HPV infection and cervical lesions (Wu et al., 2022). The decrease in *Lactobacillus* may lead to a pro-inflammatory environment, increasing the expression of HPV E6 and E7 genes and malignant cell proliferation (Kyrgiou and Moscicki, 2022). In Mexican women, HPV infection leading to cervical squamous intraepithelial lesions (SIL) is mainly associated with changes in VMB composition (Nieves-Ramírez et al., 2021). SIL patients showed increased quantities of *L. jensenii* and *L. iners* (Gomez Cherey et al., 2023). Additionally, *L. crispatus* (CST I) and *L. gasseri* (CST II) were most common in HPV-negative women, while CST III and IV were associated with HPV infection and the development of CC (Audirac-Chalifour et al., 2016), closely related to VMB dysbiosis.

Cheng Weiye et al. used Illumina high-throughput sequencing to analyze the relationship between VMB composition and HPV infection. The results showed that *Lactobacillus* accounted for over 80% in healthy women, while HR-HPV-infected individuals exhibited increased VMB diversity, mainly characterized by an increase in *Gardnerella* and a decrease in *Lactobacillus*. Furthermore, *Chlamydia trachomatis* and *Ureaplasma urealyticum* may act synergistically with HPV in the development of CC (Cheng et al., 2020). Additionally, Zhai Qingzhi et al. investigated the VMB characteristics of 168 Chinese reproductive-age women with varying degrees of cervical lesions and HR-HPV positivity. They found that the healthy group (HR-HPV negative) was dominated by *Lactobacillus* and *Ignatzschineria*, while the disease group (HR-HPV positive) was mainly composed of *Gardnerella* and *Prevotella*. In the HR-HPV group, with the progression of cervical lesions, the content of *Lactobacillus* and *Ignatzschineria* continued to decrease (Zhai et al., 2021). These results suggest that the depletion of *Lactobacillus* and the overgrowth of anaerobic bacteria may be related to VMB dysbiosis. Additionally, vaginal dysbiosis may increase the risk of infections with pathogens such as *Chlamydia trachomatis* and *Gardnerella* (Balle et al., 2018; Di Pietro et al., 2019; Pekmezovic et al., 2019).

### 4.1 Chlamydia trachomatis

*Chlamydia trachomatis*, similar to HPV, is an obligate intracellular pathogen and a major cause of bacterial sexually transmitted diseases. *Chlamydia trachomatis* has unique biphasic developmental characteristics that aid in HPV stable invasion of the host, and it causes an estimated 128 million new infections annually (Rowley et al., 2019). *Chlamydia trachomatis* is a well-known common cause of cervicitis and urethritis, often presenting as asymptomatic infections. If left untreated, it can lead to severe reproductive sequelae such as pelvic inflammatory disease, ectopic pregnancy, tubal factor infertility, miscarriage, and preterm birth (Shaw et al., 2011). A case-control study found that women infected with *Chlamydia trachomatis* tend to have *L. iners* or multiple anaerobic bacteria dominating their cervical-vaginal microenvironment (Van Der Veer et al., 2017). Additionally, a meta-analysis reviewing the impact of *Chlamydia trachomatis* infection on VMB composition showed that individuals infected with *Chlamydia trachomatis* tend to have cervical-VMB dominated by *L. iners* or a mixture of facultative or strict anaerobes (Di Pietro et al., 2022). Furthermore, a meta-analysis that firstly evaluated the relationship between VMB and infections such as HPV and *Chlamydia trachomatis* found a positive trend between a low *Lactobacillus*-dominated VMB and infections with HPV and *Chlamydia trachomatis* (Tamarelle et al., 2019). These studies suggest that genital infections caused by *Chlamydia trachomatis* have a significant impact on the composition of the cervical-VMB.

In young, unmarried, sexually active women with multiple sexual partners who use oral contraceptives, co-infection with *Chlamydia trachomatis* and HPV may be a crucial risk factor for CC (Suehiro et al., 2021; Mangieri et al., 2023). Approximately 50%-80% of sexually active individuals can be co-infected with both *Chlamydia trachomatis* and HPV, with about half of them harboring oncogenic HPV types. This co-infection may persist silently as long as the local equilibrium of VMB, immune responses, and hormones is maintained (Gargiulo Isacco et al., 2023). Italian researchers found that women co-infected with *Chlamydia trachomatis* and HPV had higher microbiota diversity in their cervical microbiota compared to the healthy group (Di Pietro et al., 2018). Furthermore, studies have shown that women who are HPV-positive and infected with *Chlamydia trachomatis* have a higher prevalence of cervical lesions compared to HPV-negative women, which may lead to the development of CC (Mosmann et al., 2021). While a relationship between *Chlamydia trachomatis*, HPV, and CC has been discovered, further research and validation experiments are still needed to clearly define the relationship between these factors and provide a scientific basis and theoretical foundation for the prevention and treatment of CC.

### 4.2 Gardnerella

*Gardnerella*, an anaerobic pathogen, is a predominant group within CST IV and a major pathogenic bacterium in bacterial vaginosis. It is often detected at higher rates in women who are HR-HPV positive (Gao et al., 2013). A cross-sectional analysis found that HR-HPV infection rates were significantly higher in women with *Gardnerella* vaginalis-associated VMB (72.73%) compared to those dominated by *Lactobacillus* (44.72%) (*P*=0.04). *Gardnerella*, along with the *Prevotella* genus, was identified as the highest-risk combination for HPV-positive women (Lin et al., 2022). Meanwhile, a prospective longitudinal cohort study suggested a positive correlation between *Gardnerella* and CIN2-CIN3, possibly due to increased VMB diversity (Usyk et al., 2020).

Gardnerella produces sialidase (SNA), an enzyme that releases sialic acid from the terminal polysaccharides on mucous secretions and mucosal cell surfaces (Severi et al., 2007). Pathogens can adhere to cells using sialic acid and alter the normal mucous barrier and immune responses (Vick et al., 2014). The genes encoding SNA include nanH1, nanH2, and nanH3, with nanH3 being associated with SNA activity (Robinson et al., 2019). Researchers have found that nanH3 levels are higher in women with persistent cervical HPV16 infection for 12 months compared to those who cleared HPV16 (*P*=0.007) (Novak et al., 2023). Elevated SNA levels are associated with susceptibility to CC (Govinden et al., 2018). In addition, studies have shown that the prevalence of SNA-producing *Gardnerella* and *Prevotella* is higher in HR-HPV infection and CIN groups, suggesting that SNA may play an important part in the development of HPV infection to cervical lesions (Lin et al., 2022). Therefore, further research is needed to understand the mechanisms by which *Gardnerella* influences HPV infection and cervical lesions, potentially identifying biomarkers that could guide the treatment of CC.

## 5 Prevention and treatment of HPV and microbiota dysbiosis

HPV infection cannot be detected within 1-2 years after infection (Markowitz and Unger, 2023), and there are currently no effective drugs or supplements to clear HR-HPV infection, so HPV infection and related lesions are mainly effectively prevented by vaccines. Based on the viruslike particles of the major capsid protein L1 pentamers, HPV vaccines are divided into bivalent, quadrivalent and nine-valent vaccines (Markowitz and Unger, 2023). Among them, bivalent vaccine mainly protects against HPV16 and 18 (Bonanni et al., 2009). Quadrivalent vaccine protects against two HR-HPV (HPV16 and 18) and two LR-HPV (HPV6 and 11) (Bonanni et al., 2009). The nine-valent vaccine, which protects against HPV6, 11, 16, 18, 31, 33, 45, 52 and 58, prevents 97% of high-grade precancerous lesions, more effective than the quadrivalent vaccine (Joura et al., 2015; Markowitz and Unger, 2023). It is important to note that the HPV vaccine does not protect against all HPV types that can progress to CC (Lei et al., 2020). Therefore, regular cervical screening is also mandatory for those vaccinated with HPV.

In addition, studies have shown that the use of α-glucans, short chain and low molecular weight polysaccharides may eradicate HPV (Cazzaniga et al., 2022). Active Xerose Correlated Compound (AHCC, a particular mixture of α-glucans) has been reported to be effective in supporting the host immune system to clear persistent HR-HPV infection (Smith et al., 2019) and is well tolerated. Results showed that IFN-β suppression to less than 20 pg/ml was associated with an increase in T lymphocytes and IFN-γ and durable clearance of HPV infection in women receiving AHCC supplementation (Smith et al., 2022).

Currently, there are two main treatment strategies for regulating the VMB, which are probiotics and vaginal microbiota transplantation (VMT). These two methods primarily aim to promote the healthy VMB to prevent and treat diseases related to HPV infection and CC.

Probiotics, referred to by the World Health Organization as “live microorganisms” (Ebner et al., 2014), include *Bifidobacteria*, *Lactobacillus*, and *Streptococci*. They help balance VMB, enhance host immune responses, and complement standard antibiotics, thereby strengthening treatment and preventing recurrence (Sharifian et al., 2023). Verhoeven et al. found that HPV-infected patients in the probiotics group had a higher HPV clearance rate compared to the control group, with the clearance rate of HPV-related cellular abnormalities being twice that of the control group (Verhoeven et al., 2013). While probiotics have a certain positive role in treating microbiota dysbiosis, their efficacy has not met the expected results. At present, there are still many questions that need to be studied, such as whether probiotics should be used at the same time of antibiotics? What is the dose of probiotics to maintain VMB balance? Does high frequency of probiotic use increase the colonization rate of VMB?

VMT is another new method for treating microbiota dysbiosis, primarily involving the transplantation of healthy microbiota isolated from donors into the patient’s vaginal environment (Ma et al., 2019). However, VMT not only requires specific conditions in the patient’s vaginal environment but also places high demands on the health of the donor’s microbiota. If the donor’s microbiota contains drug-resistant microorganisms, hidden pathogens, or sperm, it may introduce unnecessary complications to the patient (Gargiulo Isacco et al., 2023). Therefore, the VMT method is not yet mature, and further research is needed to determine its efficacy and adverse reactions to address its shortcomings (Lev-Sagie et al., 2019).

## 6 Prospects and conclusions

VMB and host maintain their health and homeostasis through co-innate. Some studies have shown that VMB components are considered as potential biomarkers for predicting disease outcomes and individualized monitoring (Kyrgiou and Moscicki, 2022). At present, most studies are focused on the relationship between VMB, HPV infection and CC, but the underlying molecular mechanism of VMB in HPV infection and cervical disease is still challenging. Therefore, large sample longitudinal cohort studies are needed to explore the effect of VMB composition changes on HPV infection and cervical disease outcomes, and analyze its role as a microbial marker of disease, which is expected to provide reasonable targets for the development of new prevention and treatment drugs.

VMB plays a significant role in HPV infection, persistence, and clearance. The occurrence and progression of HPV infection or cervical lesions are closely related to the imbalance of the VMB, primarily characterized by a decrease in the abundance of *Lactobacillus* and an increase in the diversity of the microbiota. Understanding the composition and changes in the VMB and evaluating the potential of the VMB as a novel adjunctive biomarker for predicting HPV infection and persistence will aid in the prevention and reduction of CC incidence and enhance CC prognosis. In the future, it is imperative to explore the exact molecular mechanism of VMB in the process of HR-HPV infection and cervical lesions, and to clarify the role of probiotics, prebiotics, VMT, new antibacterial agents and biofilm disrupting agents in clinical practice, so as to restore healthy VMB.

## Author contributions

ZZ: Writing – original draft, Writing – review & editing. QM: Writing – original draft, Writing – review & editing. LZ: Writing – original draft, Writing – review & editing. LM: Writing – original draft. DW: Writing – original draft. YY: Writing – review & editing. PJ: Writing – review & editing. YW: Writing – review & editing. FW: Writing – review & editing.
